# Therapists’ Professional Roles in Guided Internet-Delivered Cognitive Behavioral Therapy in Specialized Mental Health Care: Interview and Observational Study With Health Care Professionals

**DOI:** 10.2196/94640

**Published:** 2026-07-28

**Authors:** Beate Standal, Robin Maria Francisca Kenter, Monika Knudsen Gullslett, Tine Nordgreen, Inger Lise Teig

**Affiliations:** 1 Department of Global Public Health and Primary Care, Faculty of Medicine, University of Bergen, Årstadveien 17, Block D, Bergen, Vestland, 5009, Norway; 2 Research Centre for Digital Mental Health Services, Division of Psychiatry, Haukeland University Hospital, Bergen, Vestland, Norway; 3 Norwegian Centre for E-Health Research, University Hospital of North Norway, Tromsø, Troms, Norway

**Keywords:** iCBT, digital mental health, mental health professionals, therapist, qualitative research, internet-delivered cognitive behavioral therapy

## Abstract

**Background:**

Therapist-guided internet-delivered cognitive behavioral therapy (guided iCBT) is increasingly implemented in routine mental health care to expand access to evidence-based treatments. Although the clinical effectiveness and patient acceptability of guided iCBT for common mental health disorders are well established, less is known about how introducing such digitally mediated interventions reshapes therapists’ everyday work practices and professional roles. Existing research has primarily focused on treatment outcomes, service uptake, and organizational implementation challenges, leaving therapists’ lived professional experiences and role enactment in routine practice underexplored.

**Objective:**

This study aimed to explore how the introduction of guided iCBT in routine clinical practice influences mental health care professionals’ perceptions and enactment of their professional roles, and to examine how these roles are shaped by interactions between technology and health care professionals.

**Methods:**

We conducted an exploratory qualitative study using semistructured interviews and observations with 31 health care professionals who delivered guided iCBT in specialized mental health care settings between January and December 2022. Participants were recruited through purposive and snowball sampling, and data were analyzed using reflexive thematic analysis. The study was theoretically informed by actor-network theory and sociological perspectives on professional roles, enabling a sociotechnical interpretation of how professional work is shaped through interactions between human and nonhuman actors.

**Results:**

Two overarching themes described how guided iCBT reconfigures therapeutic work. First, participants reported increased professional agency through enhanced flexibility and autonomy, including greater control over work schedules and opportunities to tailor standardized treatment elements to individual patients. Second, the therapist’s role expanded beyond traditional clinical tasks, encompassing increased administrative responsibilities (“the administrative therapist”) and expectations to actively promote and legitimize guided iCBT to patients (“the professional salesperson”). While guided iCBT enabled more flexible and individualized care, it also introduced new constraints through standardized protocols, digital platforms, and organizational expectations, resulting in professional discretion and authority being actively negotiated rather than simply reduced or maintained.

**Conclusions:**

This study offers a practice-oriented, sociotechnical account of how guided iCBT is implemented in routine care. The findings show how therapists’ roles are shaped by everyday clinical workflows, in which responsibility, decision-making, and clinical judgment are distributed among therapists, patients, digital platforms, treatment protocols, and organizational arrangements. The concept of distributed therapeutic authority offers a useful lens for understanding how therapeutic work is reorganized in digitally mediated care without implying a loss of professional expertise. Professional judgment remains central but is exercised in interaction with digital tools and standardized treatment structures. These findings underscore the importance of aligning digital systems, clinical routines, and professional roles when implementing guided iCBT to support both access to care and the quality of therapeutic practice.

## Introduction

Digital technologies have long supported remote mental health treatment, from early modalities such as letters and telephone consultations to contemporary tools like videoconferencing and mobile apps [1]. In recent years, the integration of digital mental health treatments has accelerated markedly, driven by rising demand for mental health services amid workforce shortages [2], the COVID-19 pandemic, which both necessitated and normalized digital treatment formats [3], increased technological readiness [4], and shifting societal expectations regarding the legitimization of digital health [5,6]. Within this expanding landscape, therapist-guided internet-delivered cognitive behavioral therapy (guided iCBT) has emerged as an effective and scalable treatment for common mental disorders [7,8], demonstrating high patient acceptance [9] and potential time-saving benefits for therapists [10,11]. In this study, we use *guided iCBT* to refer to the specific treatment model examined, while the broader term *digital mental health treatment* is reserved for discussions of digitalization and implementation across formats and technologies.

However, introducing digital mental health treatments into mental health services is not merely a technical or logistical process. Digital technologies can transform how care is organized socially and institutionally by changing professional boundaries, challenging established therapeutic practices, and causing ambivalence or resistance among clinicians. These processes are central to the scalability, sustainability, and real-world effectiveness of digital mental health interventions [12-14]. Research on health care digitalization suggests that technology may constrain or redirect professional discretion through standardization and routinization. For example, Petrakaki and Kornelakis [15], in their study of electronic patient record implementation, showed how new systems could limit professional autonomy, leading clinicians to resist, adapt, or develop workarounds to maintain control over their practice. Although focused on administrative technologies rather than psychotherapy, these findings underscore that digital innovations can significantly impact professional roles and daily clinical work. In this study, we explore how implementing guided iCBT imposes new demands on therapists’ professional roles. Such roles encompass expectations, responsibilities, and practices enacted and negotiated within specific institutional contexts through interactions with patients, colleagues, organizational structures, professional norms, and technologies [16-18]. A central feature of professional roles is autonomy, defined as the capacity to exercise expert judgment in complex, uncertain situations [19]. Understanding how these roles are performed during implementation is vital, as role expectations and discretionary practices can influence the adoption, adaptation, and sustained use of new interventions in routine care [14]. A growing body of implementation studies has identified recurring challenges in the operation of guided iCBT services. These include difficulties in integrating iCBT within the existing health care system, often linked to skepticism toward digital treatments [20]. For example, Folker et al [21], in a comparative case study of 5 European iCBT services, identified key challenges in implementing guided iCBT, including limited integration into existing health care systems, difficulties in achieving stable patient recruitment, and changing working conditions for therapists that require new skills and support. They emphasized that ensuring long-term sustainability is critical. Together, these findings highlight that guided iCBT implementation involves ongoing adjustments in professional roles, workflows, and service organizations. These adjustments are not only organizational but are enacted through therapists’ everyday clinical decisions and interactions with digital treatment systems, yet these microlevel role dynamics remain underexplored. Much of the existing literature has focused on role changes and service organization from an organizational or managerial perspective, providing limited insight into how therapists themselves enact, negotiate, and make sense of their professional roles in everyday interaction with digital treatment systems. Despite evidence that digitalization may affect professional boundaries and autonomy, little is known about how these dynamics unfold when guided iCBT is integrated into routine clinical practice. This study addresses this gap by examining how health care professionals perceive and enact the therapist role during the implementation of guided iCBT. Drawing on qualitative interviews and field observations from 3 Norwegian mental health clinics operating within a publicly funded health care system, we ask: *How does the integration of guided iCBT into routine mental health care influence therapists’ professional roles?*

## Methods

### Study Design

This study used an exploratory theory-informed qualitative research design based on semistructured interviews with mental health care professionals in clinics delivering guided iCBT, along with observations in these clinics. The study aimed to generate an interpretive understanding of professional role enactment rather than to estimate the prevalence of attitudes. A qualitative approach was chosen to enable an in-depth exploration of participants’ experiences, interpretations, and ways of understanding their professional work. The initial aim of this qualitative study was to investigate factors supporting the implementation of guided iCBT in specialized mental health care. Accordingly, the interview guide focused on organizational, professional, and contextual conditions influencing implementation. However, as data collection progressed, participants frequently reflected on how guided iCBT affected their professional roles. In line with inductive qualitative methodology [22,23], the analytical focus was adapted to explore these emergent themes.

### Setting

Participant recruitment and data collection took place between December 2021 and December 2022. The study was conducted within Norwegian routine specialist mental health services, where guided iCBT, implemented as part of standard care, has been available since 2013 and was offered in 15 specialized mental health clinics at the time of data collection [24-26]. The clinics varied widely in size, organization, and the degree of integration of digital services. Some had dedicated units for digital mental health treatment, whereas others relied on flexible teams that incorporated guided iCBT alongside their regular clinical duties. In several clinics, treatment teams consisted of clinicians from multiple departments who met one or more times per week to deliver the treatment. Most therapists provided guided iCBT part-time (typically 20%‐50% of their position) alongside face-to-face outpatient work. A detailed description of therapist characteristics is available in Nævdal et al [27]. This variation in organizational structure, resource allocation, and implementation maturity created a rich context for examining how the introduction of guided iCBT affects therapists’ work and professional roles. Guided iCBT in this setting consists of diagnosis-specific, module-based digital treatment programs combined with asynchronous written feedback from a therapist. Each program consists of 8‐9 modules delivered over 14 weeks. Therapists monitor patient progress using standardized symptom questionnaires and provide written feedback via a secure digital platform. Patients are referred by general practitioners or psychologists or self-refer in clinics where this option is available. After a clinical assessment by the intake team, patients are assigned to a diagnosis-specific program for depression, social anxiety, or panic disorder. Key characteristics of the guided iCBT treatment in this study are presented in Textbox 1.

Textbox 1. **Key characteristics of the therapist-guided internet-delivered cognitive behavioral therapy treatment in this study.**

**Key characteristics**

- Based on cognitive behavioral therapy
- Diagnosis-specific treatment programs for depression, social phobia, and panic disorder
- Face-to-face introductory assessment interview with a therapist before treatment
- Patients complete therapy from home with written asynchronous support from a therapist
- Patients can write messages any time, and the therapist typically answers within 3 working days
- Patients fill out symptom questionnaires weekly
- Increased support, such as phone or face-to-face consultations, when necessary
- Maximum treatment time is 14 weeks
- Assessment interview with a therapist after treatment

### Participants

Participants were recruited from mental health care professionals working in 3 of the 15 iCBT clinics using purposive and snowball sampling. Clinics were purposively selected based on prior research engagement and to capture variation in organizational structure and implementation practices. Individual participants were recruited based on their involvement in the delivery, management, or implementation of guided iCBT, ensuring relevance to the study aims. Eligibility criteria included employment at one of the participating clinics and either direct involvement in guided iCBT or work within clinics where guided iCBT was delivered or managed. No additional exclusion criteria were applied.

Recruitment was initially planned to include therapists and clinic leaders responsible for organizing and overseeing service delivery. Invitations were distributed via email through clinic leaders, and the total number of invited individuals is therefore unknown. During initial interviews, it became apparent that additional professional groups played important supporting roles in implementing and operating guided iCBT services. Recruitment was therefore expanded through snowball sampling to include supporting personnel with implementation-related responsibilities and contextual knowledge of guided iCBT. This iterative approach aligned with the exploratory qualitative design and enabled inclusion of diverse perspectives on how guided iCBT was embedded in routine care. Participation was voluntary. Among those reached, 1 participant withdrew for personal reasons, 1 did not attend without explanation, 2 clinic leaders declined to participate, and 1 leader did not respond to requests to facilitate the recruitment of therapists not directly involved in guided iCBT. Therapists were included to provide insight into daily work routines, including both iCBT and non-iCBT practice. Clinic leaders from 4 administrative levels were included to capture operational, managerial, and strategic perspectives. Supporting personnel were included to capture contextual perspectives on the organization and coordination of guided iCBT, rather than for comparative subgroup analysis.

### Assessments

In line with the qualitative design, the study did not specify outcomes or predictors a priori. No standardized quantitative outcome measures were administered. The assessments consisted of qualitative exploration of experiences with guided iCBT, its implementation in routine mental health care, and its implications for clinical practice and professional roles.

The interview guide (Multimedia Appendix 1) was developed collaboratively by the authors and included questions about experiences with guided iCBT, perspectives on guided iCBT, and suggestions for increasing its use. The guide was used flexibly to support interaction and discussion in focus groups and to allow individual participants to elaborate on issues they considered particularly relevant. Follow-up questions and prompts were used to explore emerging topics in greater depth.

Participatory observations focused on everyday clinical and organizational practices related to the delivery and implementation of guided iCBT. Observational data were documented in field notes and used to support contextual understanding of interview accounts. We approached our material with the assumption that meaning is generated through “...interpretation of, not excavated from, data” [28, p. 201], consistent with reflexive thematic analysis. Consequently, we did not define adequacy in terms of data saturation. Instead, we treated thematic sufficiency as an interpretive judgment grounded in the themes’ richness, coherence, and analytical depth [23,29]. We considered themes sufficiently developed when they provided a nuanced, detailed understanding of the data relevant to our research question, based on iterative engagement with the data and the identification of consistent patterns across interviews and observations.

### Data Sources

Data were drawn from multiple qualitative sources, including individual and focus group interviews with mental health care professionals working in clinics involved in the delivery, management, or implementation of guided iCBT, as well as participatory observations conducted in clinical and implementation-related settings. All interviews were audio-recorded and transcribed verbatim. Observations were documented through written field notes recorded during and immediately after observation sessions. Interview transcripts and field notes constituted the empirical material used in the analysis and were treated as complementary data sources. All data were anonymized before analysis.

Field observations and interview data were combined into a single, integrated dataset, with each informing the interpretation of the other. Observational field notes were used to contextualize interview accounts and inform their analysis, leading to a more nuanced understanding of how work practices, organizational routines, and material arrangements related to guided iCBT were experienced and enacted. The observational material was not intended to verify participants’ statements but to enhance the interpretive analysis by showing how experiences, such as administrative burden and workflow fragmentation, unfolded in daily clinical work. This approach allowed us to situate participants’ accounts within their organizational and material context.

### Data Collection

**Figure 1.**
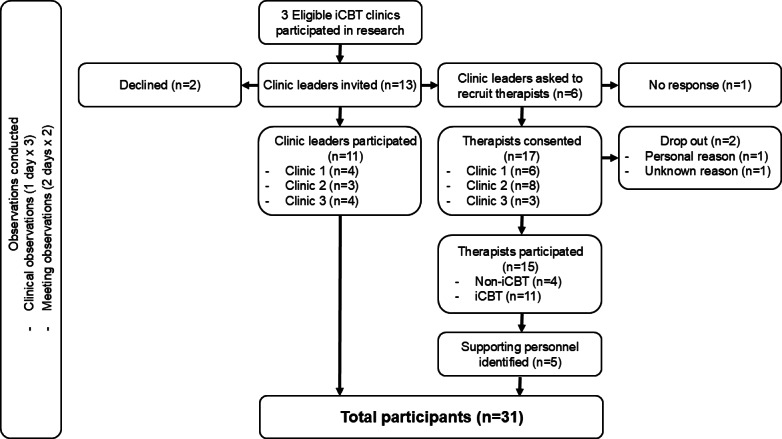
Flow diagram of participant recruitment and qualitative data collection, showing interviews and observations. iCBT: internet-delivered cognitive behavioral therapy.

Data collection comprised semistructured individual and focus group interviews, as well as participatory observations conducted in clinics delivering guided iCBT. Interviews were conducted individually or in focus groups to encourage dialogue among therapists at similar organizational levels [30]. No repeat interviews were conducted. Figure 1 presents a recruitment flow diagram outlining participant inclusion across interviews, focus groups, and observational activities.

All interviews were conducted by the first author between January and June 2022, primarily via Microsoft Teams, with two conducted by phone due to a lack of video-calling capability. Participants were situated either at their workplace or in their home office, and no individuals other than the participants were present during interviews. Individual interviews were planned to last up to 45 minutes, while focus group sessions were scheduled for approximately 90 minutes. In practice, interview durations varied, with some interviews ending earlier (a minimum of 25 minutes for individual interviews and a minimum of 51 minutes for focus groups) when participants indicated they had shared what they considered relevant. Interviews were therefore participant-led rather than constrained by fixed time limits. Interviews were conducted in Norwegian, audiotaped, and transcribed verbatim. Two transcripts were returned to participants; no comments or corrections were received.

Consistent with a reflexive thematic analysis approach, analytic rigor was supported by sustained engagement with the data and reflexive discussion among the authors, rather than by repeated interviews or formal member-checking procedures. Qualitative analysis was conducted on the original Norwegian transcripts. Translation into English occurred only at the stage of presenting illustrative quotes and was undertaken collaboratively by bilingual authors with clinical and contextual expertise to preserve meaning rather than achieve literal equivalence.

The first author conducted 1-day observations at each clinic and attended 2 cross-site meetings between May and December 2022. Observations included guided iCBT sessions, clinic meetings, and informal conversations, and aimed to capture contextual data beyond interview accounts. External meetings involved leaders and coordinators discussing implementation and collaboration across services. Table 1 summarizes the observational activities conducted across study settings.

**Table 1.** Overview of observational activities and settings.

Observation type	Setting	Purpose of observed activity	Participants involved	Duration of observation
Clinic observations (×3)	Outpatient clinics	Ordinary clinical activities	Therapists, clinic staff	3 days (one at each clinic)
Meeting 1	External venue	Discuss digital treatment across regions	Leaders from all levels	2 days
Meeting 2	External venue	Share experiences and identify barriers for implementation	Team leaders from iCBT^a^ clinics	2 days

a iCBT: internet-delivered cognitive behavioral therapy.

### Data Analysis

Data were analyzed inductively using Braun and Clarke’s reflexive thematic analysis [23,29], following 6 iterative phases: familiarization, coding, theme generation, review, refinement, and writing. The analysis focused on patterns of meaning related to professional work practices and role perceptions, rather than on frequency or consensus across participants. All interview and observational data were analyzed together as a single dataset; analyses were not conducted separately by professional groups. The first author transcribed all interviews and field notes and documented reflections throughout the analytic process. Initial coding was conducted by the first author in NVivo 14 (Lumivero) and focused primarily on semantic content, capturing therapists’ attitudes, competencies, and experiences with guided iCBT. As the analysis progressed and the analytical focus shifted toward professional roles, abductive reasoning was used to iteratively integrate empirical insights with theoretical concepts related to role and professional autonomy, particularly during theme refinement. Interpretations and theme development were discussed within the research team at multiple stages to enhance analytical depth. During coding and interpretation of interviews and observations from the clinics, we examined how features such as treatment platforms, messaging systems, documentation requirements, and monitoring tools shaped therapists’ tasks, workflows, responsibilities, and clinical discretion. This included analyzing how specific forms of work, such as administrative coordination, asynchronous communication, and treatment promotion, became necessary or expected with these technologies. An overview of early semantic codes and illustrative excerpts is provided in Multimedia Appendix 2, serving as an audit trail documenting the progression from coding to theme development. To support the transition from semantic to more latent themes, the research team wrote analytic justifications for each theme, encouraging deeper interpretation. Final themes were refined collaboratively to ensure analytical rigor and grounding in participants’ perspectives.

### Conceptual Framework

The study was informed by actor-network theory (ANT) [31,32] and perspectives from the sociology of professions literature [33,34], which conceptualize professional work as relational, negotiated, and shaped through interactions between human and nonhuman actors. These perspectives sensitized the analysis of how professional roles and practices are produced and reconfigured through everyday interactions with digital technologies and organizational arrangements.

Within these frameworks, guided iCBT is analyzed not merely as a mode of delivery but as a *sociotechnical configuration*. The digital platforms used to deliver guided iCBT, together with standardized modules and administrative procedures, are treated as active components of professional practice. These components shape how clinical tasks are organized, how responsibilities are allocated, and how professional roles are enacted.

### Ethical Considerations

The study involved human participants and was conducted in accordance with Norwegian regulations for health research. The study protocol was approved by the university’s data protection system and reviewed by the Regional Committees for Medical and Health Research Ethics (case no. 396856). The committee determined that the study was exempt from full ethical approval, as it did not involve patient data, biological material, or clinical intervention. All participants received written and oral information about the study and provided informed consent prior to participation. Participation was voluntary, and participants could withdraw at any time without consequences. No financial compensation was provided. All data were anonymized prior to analysis. Identifying information was removed from interview transcripts and field notes, and data were stored securely with access restricted to the research team.

### Reflexivity

The first author is a clinical psychologist who previously worked at one of the participating clinics. At the time of the study, the author held a university research fellowship and had no employment, clinical, supervisory, managerial, or evaluative responsibilities at any of the sites involved. Her prior familiarity with some participants was acknowledged and addressed through explicit documentation of preconceptions and ongoing reflexive discussions with coauthors throughout data generation and analysis. The potential influence of this familiarity on interpretation was continuously reflected upon within the research team. The first author had formal training in qualitative research methods and clinical psychology and conducted all interviews. Her assumptions and preconceptions regarding digital mental health treatment and professional roles were documented prior to data collection and revisited throughout analysis through reflexive memo writing and discussion with coauthors.

### Reporting Standards

This study was reported in accordance with the COREQ (Consolidated Criteria for Reporting Qualitative Research) checklist (Checklist 1) [35]. Information addressing key COREQ items is integrated throughout the Methods section, including interviewer characteristics and reflexivity, participant selection and recruitment, data collection procedures, and analytic processes. Any deviations from checklist items (eg, formal member checking or saturation reporting) reflect the study’s reflexive thematic analysis approach and are explicitly justified.

## Results

### Characteristics of Participants and Interviews

This section first presents participant characteristics and interview formats, followed by the qualitative themes. In total, 31 participants were included, representing a range of professional backgrounds and levels of experience with guided iCBT. All participants were included in the analysis, and participant characteristics are provided in Table 2. Due to the indirect recruitment procedure via clinic leaders, systematic data on refusals and reasons for nonparticipation were unavailable.

Interview formats, including the number of participants per interview or focus group session, duration, and mode of delivery, are summarized in Table 3.

**Table 2.** Participant characteristics.

	All (N=31)	Leaders (n=11)	Therapists (n=15)	Supporting personnel (n=5)
Gender				
Women	18	5	9	4
Men	13	6	6	1
Engagement with iCBT^a^				
Currently providing guided iCBT^b^	12	1	11	0
Previous clinical experience with guided iCBT^c^	4	2	0	2
No direct clinical experience with guided iCBT^d^	15	8	4	3
Occupation				
Psychologist	20	5	13	2
Nurse	6	3	1	2
Medical doctor	3	2	1	0
Other	2	1	0	1

a iCBT: internet-delivered cognitive behavioral therapy.

b “Currently providing guided iCBT” refers to participants who were actively delivering guided iCBT as part of their clinical duties at the time of data collection.

c “Previous clinical experience with guided iCBT” refers to participants who had provided guided iCBT in the past but were not delivering it during the study period.

d “No direct clinical experience with guided iCBT” indicates the absence of hands-on therapeutic delivery, although participants may have held managerial, implementation, or support roles related to guided iCBT. Some participants in leadership positions combined managerial and clinical responsibilities.

**Table 3.** Overview of interview types and characteristics.

Interview type	Number of interviews	Participants per interview	Duration (min)	Mode
Individual	16	1	25‐50	Teams/phone
Focus group	6	2‐4	51‐80	Teams

### Themes and Subthemes

Qualitative interviews and observations from 3 outpatient clinics were analyzed to explore how integrating guided iCBT into routine mental health care influences therapists’ professional roles. Two overarching themes were identified: (1) increased professional agency and (2) expansion of the therapist role. Each theme includes 2 subthemes, as summarized in Table 4.

**Table 4.** Description of themes and subthemes.

Theme and subthemes	Short description
Increased professional agency	Greater ability to exercise professional judgment within organizational and treatment-related constraints
Control over work schedule	Flexibility in organizing and structuring the workday
Autonomy to adapt the treatment	Discretion to tailor the content and structure of iCBT^a^ treatment
Expansion of the therapist role	The therapist’s role shifts and extends beyond traditional clinical work
The administrative therapist	Increased administrative responsibilities
The professional salesperson	Responsibility for promoting, motivating, and legitimizing the use of guided iCBT

a iCBT: internet-delivered cognitive behavioral therapy.

#### Theme 1: Increased Professional Agency

This theme captures how guided iCBT enabled therapists to exercise greater professional discretion within organizational structures and treatment protocols. Therapists described increased flexibility in organizing their workdays and identified opportunities to adapt the standardized digital program to better meet patients’ needs. Two subthemes illustrate how this increased agency was experienced and enacted in practice.

##### Subtheme 1: Control Over Work Schedule

Therapists consistently reported greater control over how they organized their daily work. Unlike traditional face-to-face therapy, where appointments are scheduled in advance and managed by administrative staff, guided iCBT allowed therapists to structure and adjust their consultation routines more independently. The asynchronous format enabled therapists to respond to patients between other tasks and to redistribute work throughout the day. They described handling their workload with far more flexibility, and this recently acquired flexibility was highly valued by many, as one of the team leaders emphasized:

It is a flexible way of conducting treatment...if I have 20 minutes available, I can log in and see if anyone has done anything or progressed, and then I can respond to it.

[Participant 24]

This flexibility was viewed as a significant improvement, allowing therapists to adjust the pace and sequencing of their tasks without being constrained by prescheduled face-to-face appointments. In some clinics, dedicated “digital treatment days” further supported individualized scheduling, including the possibility of longer breaks followed by more intensive work periods. One therapist described how she could follow up with 18 patients, attend a meeting, and conduct a face-to-face consultation within the same day, illustrating how guided iCBT supported a flexible work schedule and rhythm. However, therapists noted challenges associated with this flexibility. Face-to-face patients were often prioritized because they were physically present and perceived as having more acute needs. The asynchronous format also made it easier to postpone responses to digital patients, increasing the risk of delayed or inconsistent follow-up and care across patient groups. These findings illustrate how guided iCBT altered therapists’ daily routines by expanding control over work scheduling while simultaneously shifting responsibility onto therapists for regulating availability, prioritizing patients, and maintaining continuity of care.

##### Subtheme 2: Autonomy to Adapt the Treatment

Although iCBT’s standardized protocols limited certain clinical choices, therapists described several opportunities to tailor the treatment to individual patients. Across the treatment process, this autonomy was exercised at different points, including decisions about patient inclusion during intake, adaptations to treatment content, and flexibility in the forms of contact with patients. One therapist explained how the platform’s messaging function enabled such adjustments for patients they believed could benefit from the program:

I find that [guided iCBT] is often very useful, even though it is so standardized. Adjustments can be made because of the messaging function.

[Participant 17]

Therapists’ discretion over patient inclusion was one area where autonomy was evident. Even when patients did not strictly meet the program’s diagnostic criteria, therapists found ways to include patients they believed could still benefit from guided iCBT. In one clinic that allowed self-referral, the iCBT team managed its own intake processes, giving therapists greater discretion over patient flow. They could advise patients to return at a later stage if the program was not appropriate, with reentry arranged without additional administrative steps. This contrasted with one of the other clinics, where all referrals were routed through a central intake team, limiting therapists’ influence over patient selection. Having control over intake therefore gave therapists greater flexibility in shaping treatment trajectories.

Beyond decisions about patient inclusion, therapists also exercised autonomy in shaping the therapeutic content. They used the messaging function to tailor feedback, emphasize specific exercises, and supplement the program with additional guidance. Through these practices, therapists adapted standardized content to better align with individual patient needs.

Therapists further described how they modified the treatment content when they perceived the program to reflect an earlier form of cognitive behavioral therapy. To address this, they incorporated elements from newer therapeutic approaches, such as metacognitive therapy or acceptance and commitment therapy, either through written messages or during supplementary face-to-face sessions. Together, these practices illustrate how therapists exercised professional judgment to reinterpret and extend standardized treatment content.

Therapists also described autonomy in adapting the form and intensity of contact during treatment. Several noted that the high demand for face-to-face therapy made it nearly impossible to sustain weekly patient contact. By contrast, guided iCBT enabled more frequent interaction, often multiple times per week, which therapists described as more consistent with what they considered good therapeutic practice:

The treatment courses that have really worked are the ones where I have maintained a certain amount of contact with the patients, for example, by SMS and telephone as well.

[Participant 27]

Although both leaders and therapists initially assumed that communication would remain confined to the digital platform, communication practices gradually expanded as therapists gained experience and asserted greater discretion in how contact was tailored to patients’ needs. One leader noted that phone consultations had only recently been introduced, despite the program having been in use for 7 years.

Taken together, these findings show that therapists’ autonomy within guided iCBT was exercised across multiple points in the treatment process. While standardized protocols structured the treatment, therapists retained discretion over patient inclusion, treatment content, and the form and intensity of contact. Through these adjustments, therapists enacted a more flexible and personalized clinical practice, demonstrating how professional autonomy persisted despite the standardized and digitalized nature of guided iCBT.

### Theme 2: Expansion of the Therapist Role

This theme captures how the introduction of guided iCBT expanded the therapist’s role beyond traditional clinical activities. Therapists described that delivering guided iCBT involved increased engagement in administrative activities as well as promotional tasks. These activities were closely linked to the use of digital platforms and institutional expectations associated with the introduction of guided iCBT. Two subthemes illustrate how this expansion was experienced and enacted in practice.

#### Subtheme 1: The Administrative Therapist

Participants described a substantial increase in administrative responsibilities associated with guided iCBT. These responsibilities were added to their ordinary routine tasks. During observations at one of the clinics, a therapist shared a task list comprising 27 distinct work processes related to patient management. Fourteen of these were mandated by official guidelines or the electronic health record system, including documentation requirements linked to reimbursement. Notably, 4 tasks existed solely because of digital mental health treatment, such as coding the treatment as digital in the electronic health record. Therapists described these additional tasks as further increasing the administrative workload.

Most therapists found the digital platform to be cumbersome and time-consuming to use. They described it as difficult to learn, unintuitive to navigate, and poorly designed for quick access to patient information. This increased the time spent on nonclinical work, as they had to do “…a lot of clicking in that program” [Participant 26], rather than engaging with patients. Because the platform was not integrated with the electronic patient record system, documentation had to be duplicated manually across systems. Therapists described these activities as time-consuming, dissatisfactory, and unnecessary, and several therapists emphasized that such platform-related work competed directly with the time allocated for patient interaction:

We often do the job of a computer...if I didn’t spend time on the [double work], then I could probably have three times as many patients.

[Participant 27]

From the therapists’ perspective, these tasks were experienced as technical obligations rather than therapeutic work, and they increasingly structured the focus and rhythm of their everyday work.

These interview accounts were further enriched and contextualized by observational data, which offered insight into how tasks were coordinated during daily clinical routines. Observations revealed that therapists often switched between multiple digital systems to access patient information, document sessions, and track treatment progress. This workflow involved navigating between the iCBT platform and the electronic health record, often resulting in duplicate information across systems. Therapists used dual monitors to keep both platforms open, frequently switching between them to ensure accurate data transfer. Additionally, one therapist created a personal Microsoft Excel checklist to monitor administrative tasks and ensure that procedural steps were followed correctly.

Such coordination across digital systems contributed to fragmented workflows, as therapists often shifted attention between platforms to manage documentation and maintain data consistency. This reduced the time available for direct patient interactions. Overall, these observations highlight how administrative and technical tasks are integrated into everyday clinical practice, actively influencing the organization, pace, and focus of therapeutic work.

Concerns about increased tasks for therapists were also articulated in interviews at the leadership level. One leader expressed unease that iCBT therapists were increasingly engaged in tasks outside their clinical expertise:

The leaders of the iCBT team have spent an awful lot of time on contractual documents and things that I think lawyers should have taken care of and not frustrated therapists who can’t do this.

[Participant 11]

The introduction of the iCBT program entailed several new areas of responsibility, such as dissemination work, agreements and coordination across health regions, risk analysis, national approval processes, and guidance for other clinics. With no dedicated staff for these functions, such responsibilities were often allocated to therapists.

These accounts and observations illustrate how the implementation of guided iCBT has increasingly expanded therapists’ duties beyond their traditional clinical work. In this context, therapists were positioned as *administrative therapists* who were expected to maintain both therapeutic processes and the administrative and technical functions of the digital treatment service.

#### Subtheme 2: The Professional Salesperson

A recurring theme was the new expectation for therapists to actively promote guided iCBT, a role several participants described using the metaphor of being “salespersons.” Therapists, along with general practitioners, were now expected to inform both colleagues and patients about guided iCBT, presenting it as a viable treatment option, and motivating patients to engage with the program. These activities became integrated into routine clinical work, closely linked to the introduction of guided iCBT. Responsibility for promoting guided iCBT largely fell to individual therapists. As a result, whether patients learned about this treatment option often depended on the therapist’s enthusiasm, familiarity, and prior experience with guided iCBT. Therapists emphasized that the way the treatment was presented, or “sold,” strongly influenced patients’ willingness to engage. One therapist from a clinic that offered guided iCBT, but not using it personally, reflected on the importance of actively promoting the treatment:

How the therapist who has recommended the treatment sells the treatment affects what patients choose.

[Participant 6]

Participants also noted substantial differences in referral patterns. Some general practitioners and therapists frequently promoted guided iCBT, while others remained skeptical and rarely or never referred patients. One leader emphasized that “one promotes what one believes in” [Participant 26]. These variations influenced the information patients received about their treatment options and were shaped by individual therapists’ personal attitudes toward guided iCBT. At the same time, technology itself influenced therapists’ perceptions and willingness to promote the treatment. Many participants described the program as outdated and difficult to use, noting that its poor interface reduced both patient appeal and therapist engagement.

Several emphasized that improving the program’s design and usability was essential to recommending it. Participants repeatedly used terms like “attractive” and “looks good” when describing how the program’s visual and practical quality should ideally be. Therapists noted that the guided iCBT program had remained unchanged for a decade and no longer matched modern technological solutions. The perceived “old-fashioned” appearance of the programs shaped therapists’ willingness to recommend them and made promotion difficult, as one participant noted, “if you’re ashamed of how it looks” [Participant 14]. Many participants expressed discomfort promoting a treatment they perceived as unsuitable or unappealing. Several described adopting a strategy of “sitting quietly in the boat,” in response to negative patient feedback about the guided iCBT program’s visual appearance. During observations at one clinic, a manager similarly questioned the eagerness to implement the digital treatment program, criticizing it for having been introduced without sufficient consideration of whether local units were prepared for or willing to actively promote the program. Despite these concerns, most therapists delivering guided iCBT accepted responsibility for promoting it as part of their role, even though some questioned the rationale for recruiting additional patients when clinics were already overstretched. At the same time, many recognized the benefit of engaging patients earlier in their illness trajectory, noting that early intervention could help reduce the long-term burden on services.

These findings on shifting expectations illustrate how the therapist’s role gradually expanded to include responsibility for legitimizing and promoting digital treatment, specifically guided iCBT. This promotional work was largely taken for granted as part of clinical practice, contributing to positioning therapists as central actors in sustaining the visibility and uptake of digital treatment, an emerging role we identify as the *professional salesperson*.

The themes and subthemes highlight how therapeutic authority manifests in guided iCBT, showing how clinical judgment, responsibility, and decision-making are organized in practice. This authority is not held solely by the individual therapist nor entirely by the digital platform. Instead, it is reconfigured and shared within a sociotechnical network that includes therapists, patients, digital platforms, standardized treatment modules, monitoring tools, and organizational structures. While therapists maintain clinical judgment, their role is shaped by platform structures, institutional expectations, and patient engagement in self-guided tasks and symptom reporting. Thus, therapeutic authority emerges from ongoing interactions within this network, reflecting a form of distributed authority rooted in everyday clinical practice.

## Discussion

### Principal Findings

This study examined how introducing guided iCBT influences therapists’ professional roles in routine mental health care. Using reflexive thematic analysis, we identified 2 overarching themes. First, therapists experienced increased professional agency, including greater flexibility in work schedules and opportunities to adapt standardized treatment elements to individual patients. Second, therapists’ roles expanded beyond traditional clinical activities to include administrative responsibilities and expectations related to promoting guided iCBT. These changes were not perceived as purely technical adjustments but as shifts in how therapeutic responsibilities, judgments, and professional roles were enacted in everyday practice.

These findings demonstrate that digital mental health treatments such as guided iCBT do not simply extend existing clinical practice. They both support individualized care and constrain professional discretion through standardization and new organizational expectations [36]. Digital mental health treatment systems therefore influence both how therapy is delivered and what it means to be a therapist [37].

These findings align with and extend previous implementation research on guided iCBT. Building on Folker et al’s [21] identification of implementation challenges and changing working conditions in guided iCBT services, the present study contributes insight into how these changes are enacted in therapists’ everyday practice through active negotiation with digital platforms, treatment protocols, and local organizational contexts. Consistent with prior implementation research, therapists in the present study reported substantial role changes, including increased standardization, new coordination demands, and the expansion of nonclinical tasks [36]. Where no dedicated support roles were in place, therapists took on these functions, resulting in hybrid clinical-administrative roles.

However, whereas much implementation research has framed these developments mainly as organizational or managerial challenges [20], our findings suggest that role changes are also shaped by everyday clinical work. Drawing on a sociotechnical perspective (ANT), the study shows how role transformations unfold through ongoing interactions among therapists, digital platforms, treatment protocols, and institutional expectations, pointing to a reconfiguration of professional autonomy in digitally mediated care.

### Digitalization and Professional Autonomy

Concerns that digital standardization may erode professional autonomy are well-documented [12,15,36-38]. This study offers a more nuanced account. Participants described how asynchronous communication and flexible scheduling enhanced therapists’ autonomy, whereas standardized treatment structures introduced uncertainty about permissible clinical practices. These situations illustrate how standardized digital formats can blur professional discretion, requiring therapists to actively reassert their clinical judgment [39]. This dynamic aligns with ANT’s relational understanding of agency as emerging through interaction rather than residing solely in individuals.

This relational understanding of agency becomes particularly visible when examining how therapists describe adapting guided iCBT in everyday clinical work. Importantly, therapists’ descriptions of “altering” or supplementing the program did not involve modifying core treatment modules or deviating from the formal structure of guided iCBT. Instead, adaptations concerned how content was framed, how patient questions were addressed, and how broader therapeutic repertoires were mobilized through written feedback or supplementary contact. Although therapists described various ways they adapted guided iCBT, these practices are analyzed as expressions of how professional responsibility and clinical judgment are negotiated within the constraints of standardized digital treatments. Such adaptations did not alter the program but, from a sociotechnical perspective, reflect an ongoing process in which professional roles are enacted and reconfigured through relationships between nonhumans (the platform, organizational demands) and humans (professionals and patients), contributing to the redistribution of therapeutic authority observed in this study. Importantly, this study did not assess treatment fidelity or patient outcomes; therefore, these practices should be understood as part of therapists’ role negotiations within a sociotechnical system, rather than as evidence of intervention integrity or clinical effectiveness. This tension between discretion and constraints echoes findings from Petrakaki et al [38], who describe how digital technology can simultaneously drive processes of deprofessionalization, by constraining clinical autonomy, and reprofessionalization, by enabling clinicians to reinterpret and reclaim their professional discretion. In the present study, therapists eventually creatively adapted communication practices to align guided iCBT with clinical and ethical standards. This gradual expansion illustrates how autonomy was not embedded in the initial design of guided iCBT but emerged over time through therapists’ ongoing negotiation of clinical needs, technological possibilities, and organizational expectations. In doing so, they did not simply preserve professional autonomy but actively reshaped their therapeutic role within a digitally mediated care context.

### Administrative Burden and Invisible Work

Despite expectations that digital mental health treatments would streamline clinical work [11], the findings of this study suggest a more ambivalent picture. Therapists described an increase in administrative responsibilities associated with guided iCBT, as these digital systems often generated additional work rather than reduced duplication. Much of this work took the form of what participants described as “invisible work”: tasks that were essential to care delivery but not formally recognized, compensated, or reflected in job descriptions. This observation resonates with prior research on hidden labor embedded in digital health systems [40,41]. These findings are consistent with studies linking documentation demands to increased workload and burnout among health professionals [42,43].

Therapists’ accounts indicated that these administrative demands extended beyond their formal clinical training and competed directly with therapeutic work. These interview accounts were supported by observational data showing fragmented workflows, frequent switching between digital systems, and extended time spent managing procedural and documentation requirements. By displacing time and attention from direct clinical interaction, this expansion of administrative labor may therefore have implications for the relational dimensions of psychotherapy. Processes such as relational engagement and corrective emotional experiences have long been emphasized as central therapeutic mechanisms [44,45]. However, these processes may be more difficult to sustain in asynchronous and text-based treatment formats, particularly when therapists’ time and attention are increasingly directed toward administrative tasks. Structured expectations regarding responsiveness in guided iCBT, such as defined timelines for feedback, further complicate efforts to maintain relational continuity. While such structures may support patient engagement and treatment continuity, they also introduce additional demands on therapists’ time and attention. Although cognitive behavioral therapy emphasizes fostering independence and self-efficacy [46], questions concerning dependency processes associated with ongoing expectations of responsiveness fall outside the scope of this study. Overall, the findings highlight the need to consider how administrative demands are organized in relation to core therapeutic work in guided iCBT.

### Therapists as Salespersons: The Rise of Consumer Logic

A notable finding was therapists’ frequent use of marketing-oriented metaphors, such as “selling” guided iCBT or making it “attractive.” These metaphors reflected not only engagement strategies but also a perceived responsibility to legitimize and promote digital mental health treatments. This expectation aligns with broader shifts toward consumer-oriented logics in health care, where patients are increasingly positioned as consumers and services are framed as products [40]. Within this context, therapists felt responsible not only for delivering care but also for presenting guided iCBT favorably, particularly when patients expressed ambivalence or when program limitations were evident. This redistribution of promotional responsibility from institutions to individual therapists is consistent with analyses showing how market-driven pressures reshape professional roles and extend responsibilities beyond traditional therapeutic boundaries [47].

Taken together, these findings suggest that consumer-oriented logics introduce ethical tensions between care-oriented professional values and expectations to promote guided iCBT as a service, sometimes redirecting attention away from relational aspects of care [36,37]. Although not always articulated by therapists as an ethical concern, the shift from healer to service promoter can be analytically understood as ethically consequential, given clinicians’ role in shaping how treatment options are presented and legitimized. In this context, an additional concern arises when clinicians are expected not only to inform patients about treatment options but also to actively encourage uptake of a specific service. This may create tensions, as patients’ treatment choices may be subtly influenced by institutional priorities alongside clinical judgment. Several therapists described discomfort with promoting programs they themselves perceived as outdated or unappealing, highlighting potential conflicts between professional judgment and organizational expectations. These findings point to the need for future implementation research to examine how guided iCBT is presented to patients within shared decision-making processes, particularly when clinicians are simultaneously expected to promote uptake of the service.

More broadly, these tensions can be understood within processes of neoliberal commodification of health care, in which treatments are increasingly framed as products and responsibility for uptake is shifted from institutions to individual clinicians [34]. From this perspective, therapists’ discomfort reflects not individual ethical failings or resistance to innovation, but structural frictions between care-oriented professional values and consumer-oriented logics that shape digital mental health services [34,36].

Importantly, these developments do not reflect a simple loss of professional autonomy. Instead, therapists’ autonomy was both supported and constrained, while authority over the conditions of therapeutic work became increasingly distributed across digital platforms and organizational arrangements [18]. This points to a reconfiguration of therapeutic authority rather than merely a modification of professional autonomy.

### Reconfiguring Therapeutic Authority in Guided iCBT: An ANT Contribution

Building on the preceding analysis and drawing on ANT [31,32], our findings suggest that introducing guided iCBT is associated with a reconfiguration of therapeutic authority in routine mental health care. Authority is not located within the individual therapist but is redistributed across a network of human and nonhuman actors, including therapists, patients, digital platforms, standardized treatment protocols, symptom-monitoring tools, and organizational arrangements. From this perspective, guided iCBT can be understood as more than technical support for therapy delivery; it is a sociotechnical configuration that actively shapes how professional judgment and authority are enacted in practice.

At the same time, the findings show that therapists did not give up clinical authority. They retained important elements of professional discretion, for example, by adapting communication styles, tailoring feedback, and supplementing standardized content. However, their work unfolded within platform-defined structures, such as predefined modules, automated assessments, and asynchronous timelines. Therapeutic authority therefore emerged through ongoing negotiation within this sociotechnical network, positioning therapists as coactors rather than sole decision-makers. We conceptualize this configuration as distributed therapeutic authority, in which professional judgment remains central but is enacted in interaction with technological systems and organizational constraints.

The findings also point to a partial redistribution of authority toward patients. Through self-paced engagement, completion of assignments, and regular symptom reporting, patients helped shape the timing, intensity, and form of care. While such practices are often framed as patient empowerment [40,48,49], the present study suggests that this redistribution of authority to the patient also plays a central role in reshaping the therapist’s role and authority. Although the study focused on therapists’ experiences of this reconfiguration, future research should examine how patients perceive and enact these shifts in authority and how such shifts may influence therapeutic relationships over time.

These observations support the concept of *distributed therapeutic authority* as a way to capture how professional roles are reconfigured in guided iCBT. By introducing this concept, the study demonstrates how the combined framework of ANT and the sociology of professions can explain how professional authority is negotiated and redistributed through interactions among clinicians, technologies, and organizational arrangements.

### Strengths and Limitations

A key strength of this study is its use of reflexive thematic analysis grounded in therapists’ everyday practices in routine care. This approach enabled the examination of role hybridization that is often overlooked in outcome-focused research. The use of a combined framework of ANT and the sociology of professions further strengthened the analysis by illuminating how technologies, practices, and professional roles are coproduced in digitalized treatment settings. However, some limitations should be noted.

First, all data collection and early phases of analysis were conducted by the first author, who previously worked in one of the clinics. While this familiarity may have facilitated access and openness, it may also have influenced participant responses and data interpretation. Reflexive practices and systematic documentation were used to enhance transparency; however, involving multiple researchers in data collection or early coding could have strengthened credibility.

Second, participants were recruited from services that had already adopted guided iCBT, which may have resulted in an overrepresentation of favorable views of guided iCBT. This may also have led to an underrepresentation of clinicians who were strongly opposed to guided iCBT or who disengaged from its implementation, whose perspectives may be particularly relevant for understanding resistance to professional role transformation. Including professionals with limited experience of, or skepticism toward, guided iCBT could have provided broader insight into barriers to adoption. The analysis focused on shared patterns across multidisciplinary teams rather than profession-specific comparisons, due to small subgroup sizes and confidentiality concerns. Larger studies could examine differences across professional groups in more detail.

Third, patient perspectives were not included. As a result, the findings cannot address how therapists’ reported role changes are perceived by patients or how shifts in responsibility and authority affect therapeutic relationships, patient autonomy, or treatment outcomes over time.

### Implications for the Implementation of Digital Mental Health Treatment

Although therapists did not explicitly describe role changes as implementation barriers, the findings suggest that guided iCBT reshapes professional practice in ways that may influence adoption, engagement, and sustainability. While guided iCBT is often promoted as a scalable and resource-efficient solution, this study suggests a more complex picture. Rather than reducing clinical labor, guided iCBT redistributes work and responsibility among therapists by increasing administrative tasks, coordinating across digital systems, and expanding expectations related to patient engagement and program promotion.

From an implementation perspective, these findings raise questions about how coordination and support work should be organized in guided iCBT services. When such tasks are not explicitly allocated or supported at the organizational level, they tend to accumulate within the therapist role, contributing to role strain and hybridization. Implementation strategies should therefore pay closer attention to how nonclinical and coordinative tasks are distributed, recognized, and supported within digital care models.

One potential approach is to introduce dedicated support roles, such as digital navigators, that focus on coordination, onboarding, and technical tasks rather than embedding these functions within clinical roles [50-52]. Such arrangements may have implications for staffing models, training requirements, accountability structures, and cost calculations, and warrant further empirical evaluation.

Importantly, much of the work associated with digital interventions remains poorly visible at the organizational level and is often excluded from efficiency assessments, suggesting that claims about efficiency and scalability rely partly on the redistribution and partial invisibility of clinical labor. Implementation efforts should therefore approach efficiency not as an inherent property of digital interventions, but as contingent on sociotechnical design choices, workload allocation, and the extent to which redistributed work is acknowledged and supported.

Taken together, these findings indicate that implementation processes should account for how digital mental health treatments reshape professional autonomy and clinical judgment. At the same time, it is essential to preserve the flexibility valued by therapists while addressing potential risks associated with role ambiguity and workload strain. In addition, prioritizing treatment programs that minimize administrative burden and “invisible” work may support engagement and sustainability [41,42,53]. At the organizational level, explicit structures to support coordination, dissemination, and technical problem-solving may help redistribute nonclinical tasks and enable therapists to focus on core therapeutic work [50-52].

### Overall Contribution and Interpretation

This study contributes to the digital mental health literature by providing a practice-level and sociotechnical perspective on how daily professional practices support guided iCBT in routine care. It complements existing research, which is largely focused on treatment outcomes and uptake. By understanding role transformation as a sociotechnical process embedded in clinical workflows and drawing on ANT, the study demonstrates how therapeutic authority, discretion, and responsibility are distributed among therapists, patients, digital platforms, treatment protocols, and organizational structures.

The concept of *distributed therapeutic authority* provides a clinically relevant framework for understanding how therapeutic work is reorganized in digitally mediated mental health care without assuming a loss of professional expertise. Instead, professional judgment remains central but is enacted in close interaction with digital tools and standardized treatment structures. These findings have practical implications for the design and implementation of guided iCBT services, highlighting the importance of aligning digital infrastructures, clinical routines, and professional roles to ensure that expanded access to care does not come at the expense of professional autonomy or therapeutic quality.

### Conclusions

This study shows that implementing guided iCBT reshapes therapists’ professional roles in routine mental health care by redistributing authority, responsibility, and work among clinicians, patients, and digital treatment systems. While guided iCBT can enhance flexibility and support individualized care, it also challenges assumptions about efficiency and scalability by introducing new administrative, coordinative, and promotional demands for therapists. We conceptualize these changes as a form of *distributed therapeutic authority*, in which clinical judgment remains professionally grounded but is enacted in interaction with structured treatment programs, standardized protocols, and patient self-management practices. By providing detailed, context-sensitive accounts of therapists’ everyday work, this study contributes to a more nuanced understanding of the implementation of digital mental health treatments and highlights the need to attend to the reorganization of professional roles and responsibilities in digitally mediated care.

## Supplementary material

10.2196/94640 Multimedia Appendix 1 Interview guide.

10.2196/94640 Multimedia Appendix 2 Preliminary codes and illustrative quotes concerning the therapist role.

10.2196/94640 Checklist 1 COREQ 32-item checklist.

## Abbreviations

ANT: actor-network theory
COREQ: Consolidated Criteria for Reporting Qualitative Research
guided iCBT: therapist-guided internet-delivered cognitive behavioral therapy
