# Supplementary material for: Therapists’ Professional Roles in Guided Internet-Delivered Cognitive Behavioral Therapy in Specialized Mental Health Care: Interview and Observational Study With Health Care Professionals
Source: J Med Internet Res. 2026 Jul 28;28:e94640. doi: 10.2196/94640 (PMC13411433; doi:10.2196/94640)
Supplement: Multimedia Appendix 1 [file jmir-v28-e94640-s001.docx]

## Multimedia Appendix 1: Interview guide

1. Can you tell me about your experiences with guided iCBT?

a. Can you exemplify how it is used in your organization?

2. What are your thoughts about providing guided iCBT?

a. Is it a tool that can be used? How and for whom?

3. If we take for granted that guided iCBT should be used; What do you think it takes for this tool to be used and be recognized and considered equal to other treatment methods?

a. What does it take in your daily work?

b. What does it take in (name of workplace)?

c. What does it take in (name of local health trust)?

d. What does it take in (name of regional health trust)?

e. What does it take on a national level?

4. Is there anything we haven’t talked about that you think is important regarding the introduction of guided iCBT?

5. Please mention three things that you think could make guided iCBT more widely used as a treatment method.
