# Supplementary material for: Therapists’ Professional Roles in Guided Internet-Delivered Cognitive Behavioral Therapy in Specialized Mental Health Care: Interview and Observational Study With Health Care Professionals
Source: J Med Internet Res. 2026 Jul 28;28:e94640. doi: 10.2196/94640 (PMC13411433; doi:10.2196/94640)
Supplement: Multimedia Appendix 2 [file jmir-v28-e94640-s002.docx]

## Multimedia Appendix 2: Preliminary codes and illustrative quotes concerning the therapist role

| **Code** | **Illustrative quote** |
| --- | --- |
| Therapist motivation | *“ICBT therapists often have a high level of personal motivation for it”*  Participant 29 |
| Digital competence | *“The stakeholders in the outpatient clinic are the ones that are the most experienced. However, the most experienced are not necessarily the ones who are most digitally competent or most curious about new media.”*  Participant 9 |
| Commitment | *“The commitment has to be present.”*  Participant 1 |
| Easier therapeutic role | *“It’s much easier for the therapist to see what the patient has actually done between sessions. Patients often find it difficult to remember: What have I done since last time? How did the task go? In this program, they’ve actually written it down—what they did, how it went, and what they thought. In that sense, the therapist gets a good overview of what’s happening at home or between contacts. In some cases, when enough information is provided by the patient, it gives a better opportunity for insight.”*  Participant 4 |
| Experience | *“One could ask whether things might have gone better if circumstances were different. I think part of the reason is that some of our therapists didn’t have much experience.”*  Participant 30 |
| Competence | *“People often assume that since you’re good at internet-based treatment, you should be able to treat everything through guided online therapy. But there are actually two areas of expertise involved here: one is guided internet therapy itself, and the other is the specific disorder or diagnosis. And therapists vary in their strengths across these areas. There are many misunderstandings—people tend to assume it’s much simpler than it really is.”*  Participant 14 |
| Conservative therapists | *“As a profession, we tend to be a bit conservative. We’re not always great at adopting new things, at least that’s how I see it.”*  Participant 24 |
| Resistance | *“I think there’s also a kind of internal resistance to the idea that we should spend less and less time with patients, and that the quality of the therapeutic relationship is gradually being reduced. There’s a concern that we’ll see patients less frequently, and that contact will become more superficial. I believe some people feel that this is the direction we’re heading in with these digital tools.”*  Participant 3 |
| Curiosity | *“I’d heard about it for quite some time, even at my previous workplace, and I was genuinely curious. Part of that curiosity came from having all these questions: How can you deliver high-quality treatment through remote follow-up? It’s a really fascinating concept. At that point, I’d also realized that if we were going to improve patient flow in an already busy hospital, something had to change. I’d already come across this treatment option, so I was genuinely keen to understand—what is it? What are the possibilities? What are the limitations? And which patients might it actually be suitable for?”*  Participant 1 |
| Therapists retain patients | *“I think that, for example in an outpatient clinic, if these patients are considered somewhat less complex than others, then it’s perfectly reasonable for therapists to sometimes prefer seeing them face-to-face [rather than referring them to guided iCBT]”*  Participant 16 |
| Therapeutic orientation | *“I think a lot of it comes down to tradition—what we understand therapy to be. The therapeutic relationship is seen as very important, and what happens in a physical meeting between two people is considered essential. It can be difficult to imagine that this could be achieved through the internet, for example. And of course, there are different therapeutic traditions, aren’t there? Some place a strong emphasis on the therapeutic relationship itself, viewing it as a key therapeutic ingredient, if you can put it that way.”*  Participant 2 |
| Belief in the treatment | *“I believe having therapists who are confident in the approach, who enjoy working with it and feel that it’s effective, is definitely a positive aspect of the service.”*  Participant 28 |
| Willingness to try | *“We actually need to dare to try, in order to gain the necessary experience. And we have to challenge the assumption—or bias—that coming to an outpatient clinic is always the better option.”*  Participant 9 |
| More challenging therapeutic role | *“I find that when a crisis arises within a digital treatment program, it’s often more difficult to manage than in a traditional outpatient setting.”*  Participant 3 |
